# New Emerging Recombinant HIV-1 Strains and Close Transmission Linkage of HIV-1 Strains in the Chinese MSM Population Indicate a New Epidemic Risk

**DOI:** 10.1371/journal.pone.0054322

**Published:** 2013-01-23

**Authors:** Jianjun Wu, Zhefeng Meng, Jianqing Xu, Yanhua Lei, Lin Jin, Ping Zhong, Renzhi Han, Bin Su

**Affiliations:** 1 Anhui Provincial Center for Disease Control and Prevention, Hefei, China; 2 Shanghai Public Health Clinical Center, Key Laboratory of Medical Molecular Virology of Ministry of Education/Health at Shanghai Medical College, Fudan University, Shanghai, China; 3 Department of AIDS/STD, Shanghai Municipal Centers for Disease Control and Prevention, Shanghai, China; 4 Hefei Center for Disease Control and Prevention, Hefei, China; 5 Department of Cell and Molecular Physiology, Loyola University Chicago Health Science Division, Maywood, Illinois, United States of America; Fundacion Huesped, Argentina

## Abstract

In recent years, the population of men who have sex with men (MSM) have become the most significant increasing group of HIV-1 transmission in China. To identify new recombinant strains and transmission patterns of HIV-1 in Chinese MSM population, a cross-sectional investigation of MSM in Anhui Province (in south-eastern China) was performed in 2011. The diagnosed AIDS case rate, CD4 T-cell counts, HIV subtypes, and origin of the recombinant strains were investigated in 138 collected samples. The phylogenetic and bootscan analyses demonstrated that, apart from three previously reported circulating strains (CRF07_BC, CRF01_AE, subtype B), various recombinant strains among subtype B, subtype C, CRF01_AE, and CRF07_BC were simultaneously identified in Chinese MSM for the first time. The introducing time of B subtype in Chinese MSM populations was estimated in 1985, CRF01_AE in 2000, and CRF07_BC in 2003; the latter two account for more than 85% of MSM infections. Notably, in comparison with B subtype infections in Anhui MSM, CRF01_AE, with the highest prevalence rate, may accelerate AIDS progression. Over half of patients (56%) infected with new recombinant strains infection are diagnosed as progression into AIDS. Both Bayes and phylogenetic analyses indicated that there was active HIV transmission among MSM nationwide, which may facilitate the transmission of the new 01B recombinant strains in MSM. In conclusion, new recombinant strains and active transmission were identified in the Chinese MSM population, which may lead to a new alarming HIV pandemic in this population due to the increased pathogenesis of the newly emerging strains.

## Introduction

As of 2011, there are 780,000 people in China infected with HIV. 59.0% of these individuals received this virus through sexual transmission: 44.3% through heterosexual transmission and 14.7% through homosexual transmission [1]. Among the 54,000 new infections estimated for 2011, heterosexual transmission accounted for 42.2% and homosexual transmission for 32.5% of cases [1]. This significant increase in homosexual transmission shows an alarming trend that is also observed in other Asian countries [2], [3], [4], [5]. Compared with the estimated 12.2% in 2007, homosexual transmission has thus become a very significant mode of transmission for new HIV infections in China [1], [5], [6], [7].

In recent years, HIV-1 infection in the Chinese MSM population has received increased attention from Chinese researchers [8]–[13]. Molecular epidemiological investigations of HIV-1 were performed in MSM from Beijing and local cities in Liaoning, Hebei and Henan provinces [8]–[9], [11]–[13]. These studies all indicated similar epidemic conditions, with multiple circulating HIV-1 subtypes (including B, CRF07_BC, CRF01_AE) in different provincial MSM populations in China, making it possible for recombinant events to potentially occur in the MSM population. However, due to the lack of systematic investigations in MSM, much remains to be determined. For example, it is not clear whether there are new circulating recombinant strains in the MSM population and exists transmission linkage among different epidemic regions derived MSM populations. Moreover, it is important to study the effects of the dynamic prevalence rate of various circulating HIV subtypes in MSM on AIDS progress in patients, which is helpful for adjusting strategies for prevention and intervention against MSM infections.

Anhui, a province located in south-eastern China and neighboring Henan in the north, is a severe HIV-1 epidemic region where, traditionally, most infections were caused by former blood donation (FBD). However, new infections are rapidly increasing through homosexual transmission among MSM [1], [6], [7]. To date, 768 infections were reported in MSM from Anhui province, with 138 of them newly reported in 2011. In this study, we collected all 138 MSM samples and analyzed the *gag* and *pol* genes.

This first cross-sectional investigation in provincial MSM populations was designed 1) to characterize the circulating HIV-1 subtypes and new recombinant strains and 2) to study subtype-related AIDS progression in the MSM population. Moreover, a phylogeographical tree was constructed to examine the transmission linkage and estimate the time of the most recent common ancestor (tMRCA) of different epidemic regions in Chinese MSM. These results will be useful for evaluating the risk of a potential new wave of HIV transmission in the Chinese MSM population.

## Materials and Methods

### Ethics Statement

The study was reviewed and approved by the local ethical review committee at the Anhui Center for Disease Control and Prevention. Written informed consent was provided from all participants.

### Study Participants and Specimens

In total, 138 newly confirmed HIV-1–positive MSM samples in 2011 were collected from 15 prefectures of Anhui. The study was conducted in a cross-sectional and anonymous way, according to guidelines of the Anhui Center for Disease Control and Prevention, which provides HIV/AIDS treatment and prevention services through education, quality services, and compassionate care to those with AIDS/HIV infection. HIV infection was confirmed in all subjects by Western blotting (HIV blot 2.2; Genelab Technologies, Inc., Singapore) and HIV/AIDS progression in all participants was diagnosed by a physician from Anhui CDC. Fresh whole blood samples were collected for CD4/CD8 T-cell counts and plasma samples were stored at −80°C for viral RNA extraction. CD4 T lymphocytes in whole blood were assessed using flow cytometry using the reagents and equipment provided by Becton Dickinson Biosciences (San Jose, CA, USA). Statistical analyses were performed using Student's *t*-test in Microsoft Excel.

### Sequencing HIV-1 *gag* and *pol* Gene Fragments

Viral RNA was extracted from patient plasma using the QIAamp Viral RNA Mini kit (Qiagen, Valencia, CA, USA). HIV-1 cDNA was obtained by RT-PCR using the TaKaRa One Step RNA PCR kit (TaKaRa Biotechnology Co. Ltd., Dalian, China), and then subjected to nested multiple polymerase chain reaction (PCR) for the amplification of *gag* and *pol* genes, as described previously [14], [15]. The portion of the *p24* and *p17 gag* region (HXB2, 836–1507 nt) was selected because it has been used previously for HIV-1 phylogenetic analysis and appeared less prone to recombination [16], [17], [18]; in contrast, the *pol* fragment (HXB2, 2147–3462 nt) is considered as a “hotspot” for recombination, particularly in BC recombinant strains, and was selected for recombinant analysis. Purified PCR products (Qiagen gel extraction kit, Qiagen) were sequenced using fluorescent dye terminators (Prism BigDye terminator cycle sequencing ready reaction kit; Applied Biosystems, Foster City, CA, USA) and an automated ABI 377 DNA Sequencer (Applied Biosystems) [13], [19].

### Phylogenetic and Bootscan Analysis of HIV-1 Sequences

To eliminate potential contamination, the resulting sequences were first subjected to an HIV-1 Blast search to compare with related reference sequences in the HIV databases (www.lanl.hiv.gov/index). The resulting *gag* and *pol* gene fragment sequences were aligned with reference sequences of HIV-1 strains of various subtypes from the Los Alamos HIV-1 database. Multiple alignments were made automatically using the Bio-Edit 7.0 software with minor manual adjustments. Phylogenetic analysis of the aligned sequences was performed using the neighbor-joining method using MEGA 5.02. In the neighbor-joining tree generated, the statistical robustness of the tree and the reliability of the branching patterns were confirmed by bootstrapping (1000 replicates). At the same time, a bootscan analysis of the *pol* gene was performed using the SimPlot 2.5 software [13], [19], [20]. Reference sequences used for the bootscan analysis included subtype A1 (92UG037), subtype B (HXB2, BK132), subtype C (95IN21068), subtype G (92NG083), CRF01_AE (05GX001), and BC (98CN009). Bootscanning was performed with a sliding window of 300 nucleotides, overlapped by 20 nucleotides, to define the recombinant structure.

### Sequencing near full-length sequences

A three-amplicon strategy was adopted to obtain near full-length sequences from samples with potential new recombination sites. The preparation of viral RNA, RT-PCR, and sequencing approach were described previously [21]. The near full-length genome sequence was assembled by overlapping the sequences of the three amplicons and merging them into one sequence as long as the two overlapping sequences were greater than 99% similarity [13], [21]. The resulting near full-length sequences were used for bootscan analyses using SimPlot 2.5, in the context of all references sequences of pure subtypes(A-K) and common recombinant strains (CRF01 to CRF15) from HIV database, which in turn indicated the most potential parental sequences of new recombinants. Based on the sequences of predicted parental strains and some background strains, the detailed recombination sites of new recombinant strains were defined with a sliding window of 300 nucleotides, overlapped by 20 nucleotides, against near full-length sequences, as described previously [13], [19], [20]. To further trace the origin of the new recombinant strains, a neighbor-joining tree was constructed using the recombinant fragments and the predicted parental strains. The new HIV sequences are accessible in GenBank from KC203088 to KC203332 and from KC183774 to KC183783.

### Transmission linkage analysis using phylogeographical trees

For phylogeographical tree analysis, China-derived *gag* and *pol* full-length gene sequences, labeled CRF01_AE, CRF07_BC and B, up to August 2012 were retrieved from the Los Alamos HIV Sequence Database (www.hiv.lanl.gov, Table 1). Moreover, Asian near full-length sequences of 01B and 01C were also retrieved from the same HIV database (Table 1). Datasets of Anhui sequences and retrieved Chinese sequences were prepared with Bio-EditV7.0 and Mega5.02, and subsequently used to construct a phylogeographical tree using the BEAST V1.6.2 package [22]–[26]. The Bayes MCMC analyses and Bayes factor test were performed as described previously [22], [25], [26]. To construct maximum clade credibility (MCC) trees, the initial 25% of trees generated were discarded as “burn-in” and the living trees per run were summarized using TreeAnnotator, implemented in the BEAST V1.6.2 package [22], [26]. All trees were examined and edited using FigTree V1.3.1 (tree.bio.ed.ac.uk/software/figtree/), which was also used to estimate evolutionary rates and dates to the time of the most recent common ancestor (tMRCA) of various nodes on the MCC tree [24], [25], [26]. Posterior probabilities for the internal nodes were calculated from the posterior density of the trees [24], [26]. Statistical uncertainty in parameter estimates is reflected by the values of the 95% highest posterior density (HPD) credible region (CR).

**Table 1:** Retrieved HIV-1 sequences from the HIV database for Bayes Analysis.

gene	CRF01_AE	CRF07_BC	B	01B*	01C*
*gag*	Yunan: 1	Yunan: 9	Yunan: 15	CN:1	MM:2
	Guangxi: 15	Guangxi: 2	Hubei:10	JP:2	CN:1
	Fujian:13	Xingjiang:5	Henan: 14	MM:2	TH:1
	Jiangsu:1	Taiwan:1	Hebei: 12	MY:15	
	Liaoning:11	Liaoning:8	Liaoning:15	TH:32	
	Hebei: 9	Hebei: 2	TH:22	ID:2	
	Henan: 12	Henan: 5	US:13#	SG:2	
*pol*	Yunan: 7	Guangxi: 2	Yunan: 1		
	Guangxi: 10	Xingjiang:5	Guangxi: 2		
	Fujian:8	Taiwan:1	Hubei:1		
	Jiangsu:1	Hebei: 1	Henan: 14		
	Liaoning:6	Henan: 4	Hebei: 2		
	Shandong: 7		TH:22		
	Hainan: 6				
	Hebei: 7				
	Beijing:7				

* Near full length sequences.

CN: China, JP: Japan, MM: Myanmar, MY: Malaysia, TH: Thailand, ID: Indonesia SG: Singapore, US: the United State.

# one from France.

## Results

### Characteristics of Study Participants in the Anhui MSM Study

**Figure 1:**
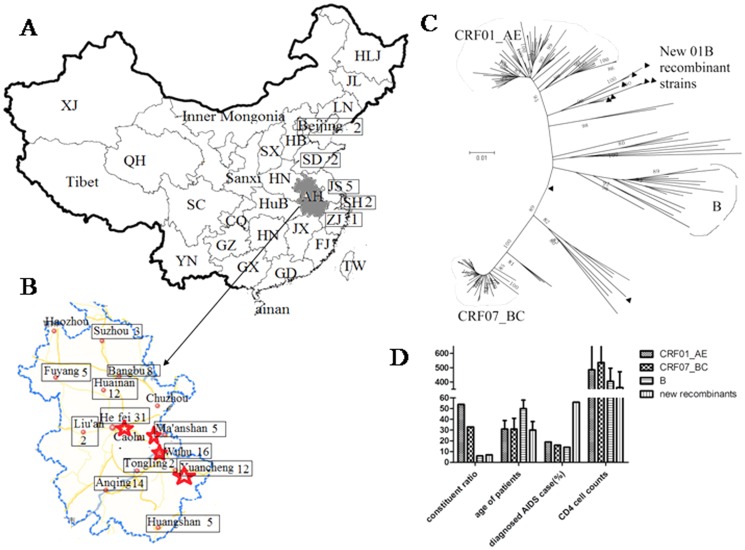
Geographical Distribution and Phylogenetic Analysis of three subtypes (CRF01_AE, CRF07_BC, B) and new recombinant strains from Anhui MSM. Box and number represent the regions with infections of Anhui MSM HIV-1, stars represent the regions with 4 new 01B strains (46,59,73,115), and triangles represent 9 new Recombinant strains. A: The geographical distribution of Anhui MSM infections in other provinces of China. B: The geographical distribution of HIV infections of MSM in Anhui province. C: Neighbor-joining tree of *pol* gene fragments from Anhui MSM infections, the reference sequence is retrieved from HIV database. D: The demographic and clinical information of participants from Anhui MSM, The data represent Mean ± SD. • The abbreviations of Chinese provinces: BJ: Beijing, HuB: Hubei, SH: Shanghai, HN: Hunan, TJ: Tianjing, GD: Guangdong, CQ: Chongqing, GX: Guangxi, HB: Hebei, HI: Hainan, SX: Shanxi, SC: Sichuan, GZ: Guizhou, LN: Liaoning, YN: Yunnan, JL: Jiling, HLJ: Heilongjiang, JS: Jiangsu, ZJ: Zhejiang, QH: Qinghai, AH: Anhui, FJ: Fujian, XJ: Xinjiang, JX: Jiangxi, TW: Taiwan, SD: Shandong, HA: Henan.

The geographic distributions of these HIV-positive samples are summarized in Figure 1A. Only a few cities were not involved in HIV-1 transmission of Anhui MSM (Figure 1B). Notably, some patients of Anhui MSM had lived and worked in other provinces where they became infected. They moved to Anhui recently and were followed by us (Table 2). Therefore, in some extent, these immigrants may facilitate the linkage of HIV-1 strains among different provincial MSM populations. Based on patient records, most (95%) of the study participants were between 18 and 45 years of age, suggesting that the majority of the labor force and sexually active people are more vulnerable to infection. Among the remaining participants, 5% were older than 45 years and none were younger than 18 years. Occupations of the study participants included farmers (25%), self-employed (13%), factory workers (20%), civil servants (10%), students (9%), migrant workers (9%), army soldiers (4%), and unknown (10%). Based on the characteristics described above, the participants recruited in Anhui are broadly representative of Chinese MSM population across the country under currently booming economical development.

**Table 2:** The information of infections who migrate between Anhui and other provinces.

Patient ID	subtype	age	Occupation	Born province	Working province	Province of first HIV-test	Province of following visit
1	CRF01_AE	25	Student	Anhui	Shanghai	Shanghai	Anhui
3	CRF01_AE	31	N/A	Anhui	Jiangsu	Jiangsu	Anhui
14	CRF07_BC	26	Sexual worker	Anhui	Zhejiang	Zhejiang	Anhui
15	CRF01_AE	27	public server	Anhui	Jiangsu	Jiangsu	Anhui
16	CRF01_AE	23	business	Anhui	Shanghai	Shanghai	Anhui
20	CRF07_BC	33	N/A	Anhui	Beijing	Beijing	Anhui
38	CRF01_AE	24	teacher	Anhui	Jiangsu	Jiangsu	Anhui
49	CRF01_AE	34	worker	Anhui	Jiangsu	Jiangsu	Anhui
79	CRF07_BC	22	N/A	Anhui	Shandong	Shandong	Anhui
91	CRF01_AE	50	worker	Anhui	Jiangsu	Jiangsu	Anhui
105	CRF07_BC	31	business	Anhui	Shandong	Shandong	Anhui
133	CRF01_AE	39	housekeeper	Anhui	Jiangsu	Jiangsu	Anhui

### Identification of HIV-1 Subtypes in Anhui MSM population

The HIV-1 genes in the patient plasma samples were analyzed by RT-PCR. We successfully obtained HIV-1 *gag* genes and *pol* genes from 131 samples of the 138 patients. A 650 bp long *gag* fragment and a 1200 bp *pol* fragment from Anhui MSM infections were used to construct neighbor-joining trees. As seen in the *pol* neighbor-joining tree (Figure 1C), there were three known Chinese circulating subtypes (CRF01_AE (72/131; 55%), CRF07_BC (44/131; 33.5%), and B (6/131; 4.5%), and nine new recombinant strains (9/131; 7%) in the Anhui MSM population (Figure 1C). It was evident that CRF01_AE and CRF07_BC were the two major subtypes likely to be co-dominating the current HIV-1 epidemic in Anhui MSM. The bootscan analysis of *pol* indicated nine new recombinant strains descendents from subtype B, CRF01_AE or CRF07_BC (Figure S1). Among the nine new recombinant strains, the *pol* gene of 4 strains (46,73 and 59,115 ) shared the similar CRF01_AE/B breakpoint sites, two strains (64, 77) showed another CRF01_AE/B recombinant model, and three strains (17, 69, 104) supposed to be the versatile products of CRF07_BC and CRF01_AE.

Based on the phylogenetic analysis results, Anhui MSM can be classified into four groups: CRF01_AE, CRF07_BC, B, and new recombinants (Figure 1D). The average age of Anhui MSM patients was 32 years old, while the age of the B subtype group was the oldest (50±8) and other three groups have close age (Figure 1D). Moreover, the CD4 cell counts of the B subtype group and new recombinant group was all less than 500 cell/µL. Notably, although there are only 9 patients, more than 50% of the new recombinant group (6/9) were diagnosed as progression into AIDS (Figure 1D), which deserved further investigation.

### Analysis of origin and recombinant structure of nine near full-length sequences

**Figure 2:**
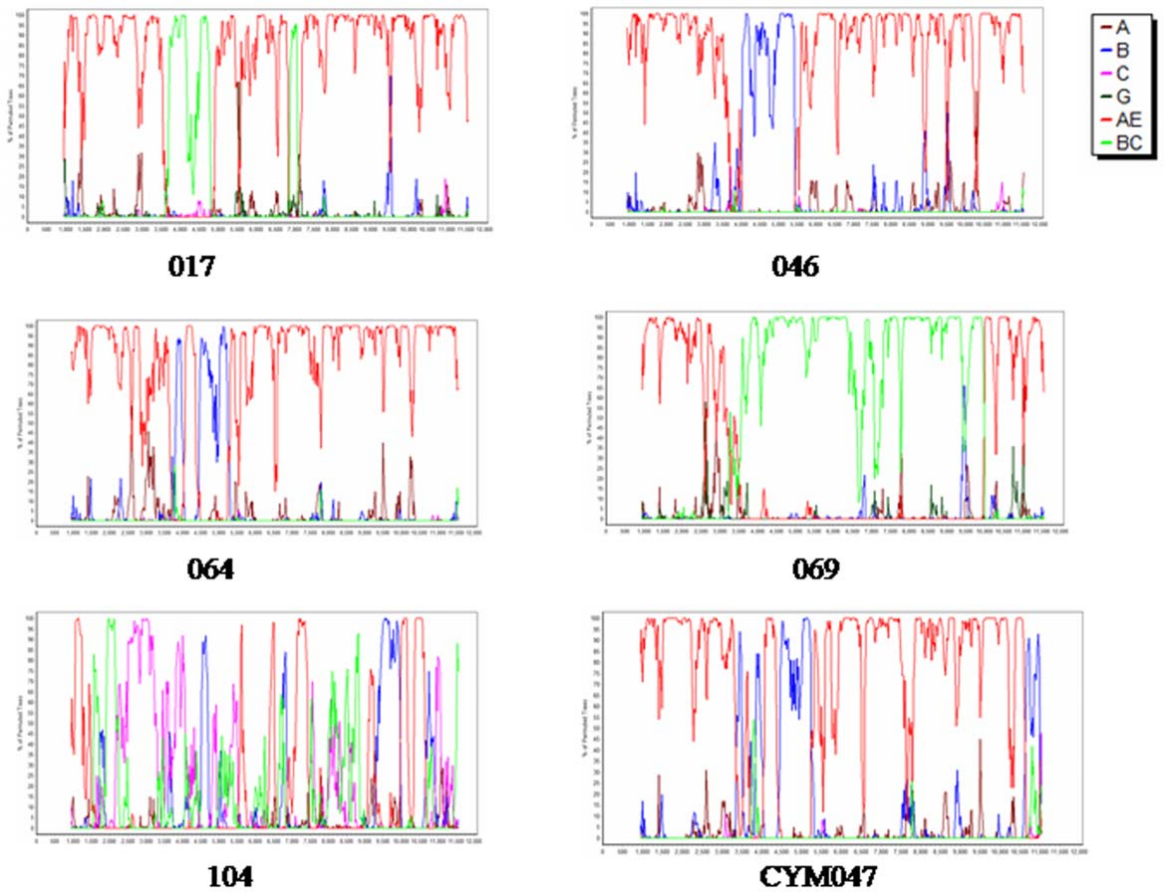
Bootscan analysis of 9 near full-length sequences. Sequences 046(from Xuanchen, XC), 059 (from Ma'anshan, MAS), 073 (from Wuhu,WH) and 115 (from Hefei, HF) showed a similar recombinant structure while sequences 64 and 77 showed a different 01B recombinant pattern. These newly identified recombinants are different in breakpoint sites from previously reported 01B strains CYM047 found in Beijing MSM. 017, 069 are the various products generated by CRF01_AE and CRF07_BC. 104 is a CPX generated by multiple HIV subtypes including CRF07_BC, C, B, CRF01_AE.

To accurately identify the recombinant structure, bootscan analysis of nine near full-length sequences generated was performed, as described previously [13], [19], [20]. In accordance with the bootscan analysis of the *pol* gene, six strains descended from CRF01_AE and B (Figure 2). Four of these strains (46, 59, 73, 115) shared one similar 01/B recombinant structure and the other two (64, 77) shared another 01/B recombinant structure, although these recombinants were all generated by insertion of B fragments into the *pol* region of the CRF01_AE backbone (Figure 2, Figure S2). From reviewing the epidemic information, there was no direct epidemic linkage among the four new 01/B recombinant strains (46, 59, 73, 115), which were isolated from four prefectures (Figure 1B: 46 from Xuanchen (XC), 59 from Ma'anshan (MAS), 73 from Wuhu (WH), 115 from Hefei (HF)) and harbored identical breakpoint sites. Further studies are required to determine if these new 01/B recombinant strains belong to CRFs or URFs. Finally, the recombination between CRF01_AE and CRF07_BC showed a complex recombinant pattern; in particular, 104 is obviously a complex (CPX) of CRF01_AE, CRF01_B and CRF07_BC subtypes.

**Figure 3:**
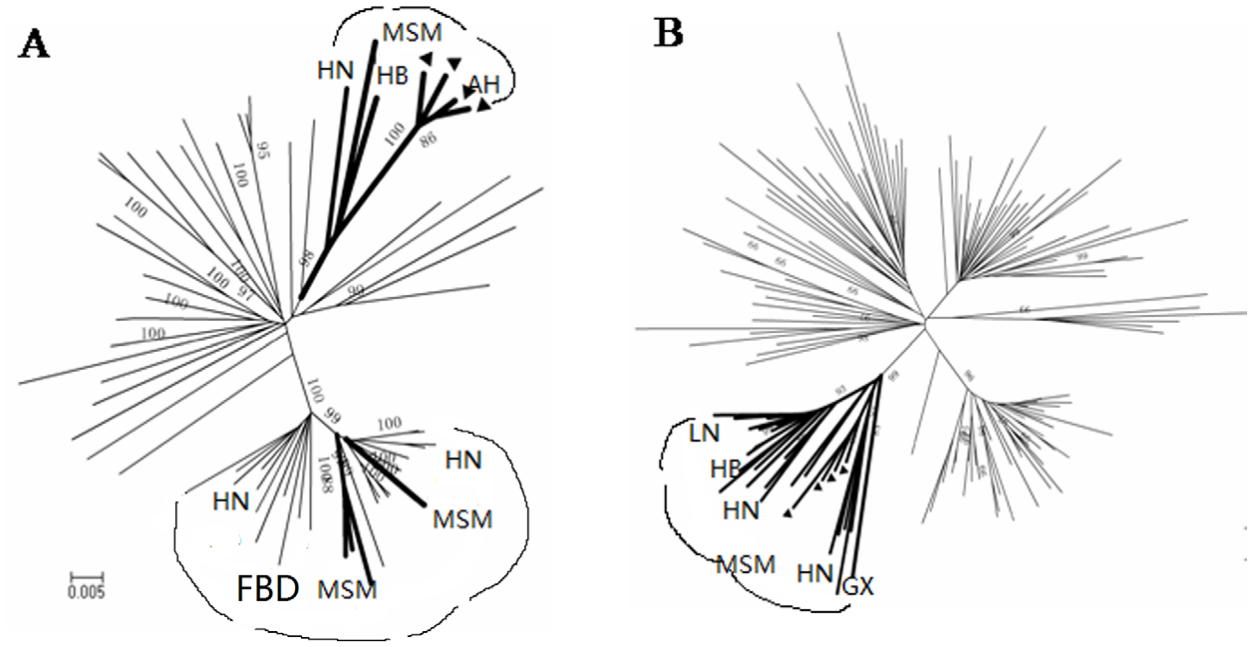
Phylogenetic analysis of new 01B CRF. A: Neighbor-joining tree of B derived fragment (covering from nt 3400 to nt 4800 HXB2) of 4 new 01B full-length sequences (46,59,73,115). Triangle represents 4 New Recombinant strains; Black and bold branches represent MSM strains formed branch. B: Neighbor-joining tree of CRF01_AE derived fragment (covering from nt 790 to nt 2292 HXB2) of these 4 new 01B sequences.

As shown in Figure 2, the region of 4 new 01B full-length sequences (46, 59, 73, 115), covering nt 3400–4800 (HXB2), was derived from a B subtype, while the rest portion was from CRF01_AE. The B fragments of these 4 01B sequences (HXB2, 3400–4800 nt) were aligned with all available Chinese B subtype strains in the HIV database and were used for a subsequent phylogenetic analysis (Figure 3). As shown in Figure 3A, the B fragment of these 4 new 01B sequences exhibited high genetic homology with B subtypes from Henan and Hebei MSM, but not Thai-B in FBDs. The CRF01_AE *gag* fragment (HXB2, 790–2292 nt) of these 4 01B sequences also formed a big cluster with MSM-derived strains (bootstrap value >95%, Figure 3B), supporting that the new 01B recombinant strains originated in MSM rather than being introduced from another high-risk population.

For purposes of comparison, we further examined the recombinant structure of all available CRF and URF of 01B and 01C sequences from the HIV database (www.hiv.lanl.gov) using a bootscan analysis. Newly identified recombinant forms from Anhui MSM differed in breakpoint sites, in contrast with all previously reported CRF or URF from Asian countries (data not shown). These results not only indicate that these new 01B recombinant strains originated from circulating CRF01_AE and US-derived B in MSM, but also revealed that there are close transmission linkages among different epidemic region-derived MSM strains.

### Transmission linkage of HIV-1 strains in MSM from different provinces

**Figure 4:**
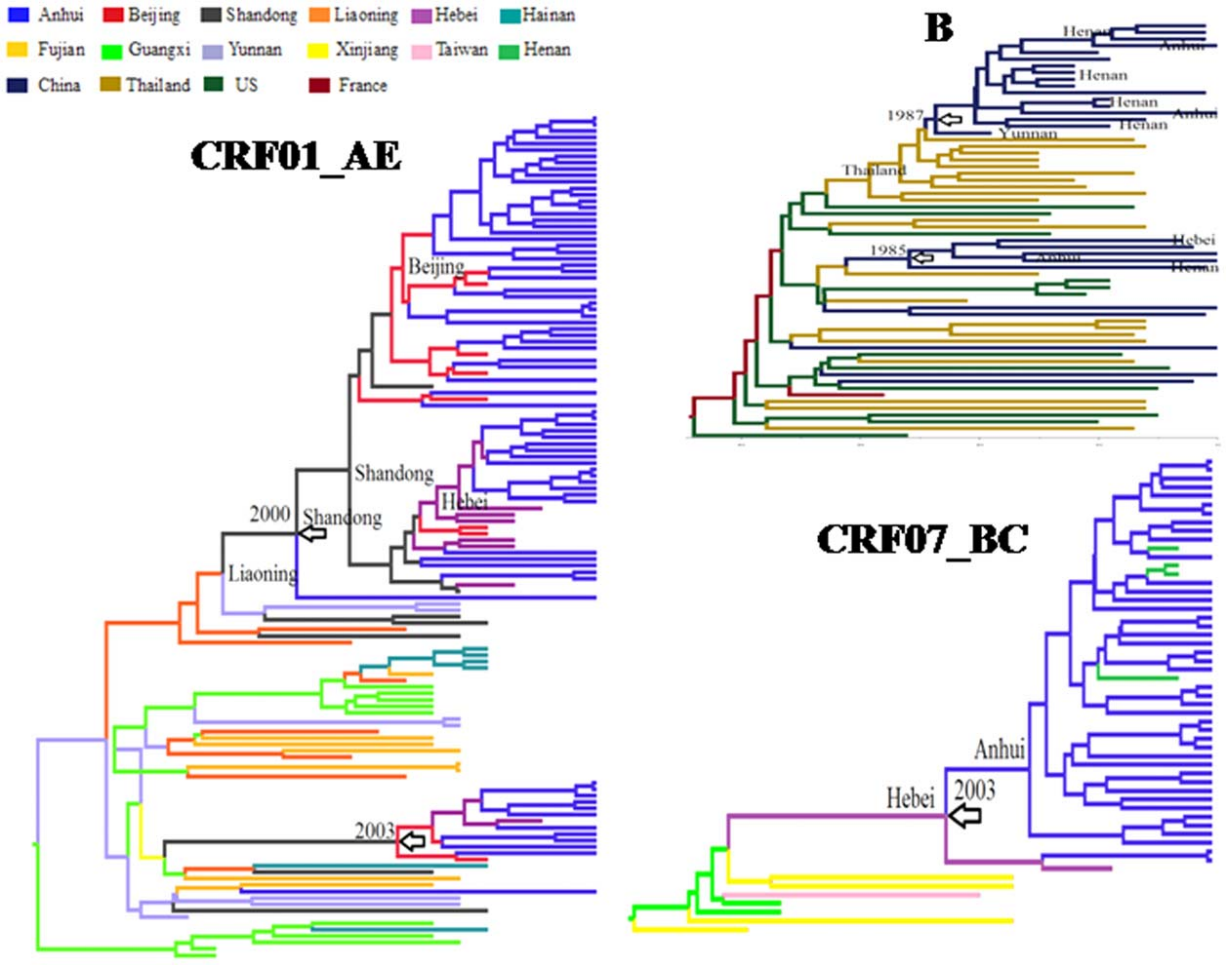
Phylogeographical tree of *pol* gene of CRF01_AE, CRF07_BC, B subtype. Ancestral geographic states were constructed using Bayesian phylogeographic inference framework implemented in the BEAST V1.6.2 package. The tree branches are colored according to their respective geographical regions. The time of most recent common ancestor (tMRCA) of clade was indicated in the node labels.

As Figure 4 shows, the phylogeographical trees of the three subtypes firmly supports that there are significant transmission linkages in provincial MSMs, indicated by the big cluster formed by all MSM strains, with high posterior probability. The Bayes Factor analysis also demonstrated significant transmission linkage among Anhui strains and strains from other provinces such as Liaoning, Hebei and Beijing (Table 3). Moreover, the phylogeographical tree revealed the various tMRCA of three subtypes in Chinese MSM and Anhui MSM.

**Table 3:** The Bayes factors test between defined locations derived CRF01_AE *pol*.

Origin	AH	BJ	FJ	GX	HA	HB	JS	LN	YN
Anhui(AH)		4.1				4.9			
Beijing(BJ)				3.2		3.3			
Fujian(FJ)				4.1			3.9		
Guangxi(GX)	4.1	6.7	6.2		8.9			4.2	
Hainan(HA)		3.3		4.8					
Hebei(HB)	11.2	7.8						3.7	
Jiangsu(JS)				4.3				3.2	3.2
Liaoning(LN)	5.1	9.2				4.2			
Yunnan(YN)	3.1		4.7				5.9	4.7	

• Bayes factors above 3 that represent statistically significant phylogeographic links between defined locations are shown.

Two distinct B groups (US-derived B and Thai-B) were observed in the Chinese MSM population: US-B and Thai-B subtype. US-B subtype was found in MSM in 1985 and is the earliest circulating strain; Thai-B was transmitted into MSM around 1987. Thai-B subtype strains from Anhui and Henan MSM cluster together (Figure 4) and show close transmission linkage with Thai-B from local FBDs. Moreover, US-derived B in Anhui MSM (around 1994) clusters together with the B subtype in Henan, Hebei, and Liaoning MSM. Obviously, the B subtype has been reduced because of the newly entering CRF01_AE and CRF07_BC in recent years, in accordance with previous reports by Wang et al. [12]. The latter two subtypes also showed different times of origin in MSM. CRF01_AE was transmitted into Chinese MSM around 2000 and transmission from Liaoning or Shandong MSM to Anhui MSM occurred around 2004. Similar to the B subtype in MSM, there were also two genetically distinct CRF01_AE strains found, which were introduced into MSM at different time points. CRF07_BC was transmitted into Chinese MSM around 2003 and into Anhui MSM in 2005. Originating from injecting drug users (IDUs), CRF07_BC in MSM formed a close cluster (Figure 4). The tMRCA of the three MSM subtypes revealed three stages of HIV transmission in MSM: the earliest transmission of the B subtype (around 1985), subsequent transmission of CRF01_AE (around 2000), and later transmission of CRF07_BC (around 2003), in accordance with the depicted age distribution of different subtype infections in Figure 1D.

The phylogeographical tree constructed with *gag* gene sequences also confirmed the above results by Bayes analysis of the *pol* gene (data not shown). Taken together, these analyses not only confirmed the close transmission linkage in MSM strains, but also revealed the origin times of these strains.

## Discussion

In recent years, MSM have become the most significant increasing HIV-1 transmission route in China. Although several investigations focusing on MSM populations have been conducted, this is the first reported systematic cross-sectional investigation of MSM covering one epidemic region (Anhui province). In accordance with the HIV epidemic in China, Anhui is a severely HIV infected region with more than 10,000 FBD infections that now faces a new challenge in the MSM population.

As an inland and labor force-exporting province, Anhui is a typical accepting epidemic region of HIV infection and transmission. Thus, the investigation of Anhui MSM not only revealed local molecular epidemiology characteristics but also partially characterized HIV infection in all Chinese MSM. The Anhui MSM patients identified may have been infected locally or in other epidemic regions, including Jiangshu, Zhejiang, Shandong, Shanghai, and Tianjing, which may provide epidemiological evidence for the tight linkage of MSM nationwide. Moreover, by analyzing the transmission linkage between Anhui MSM and other MSM, our study first indicated close transmission linkage among the different epidemic regions in Chinese MSM.

As described previously, CRF01_AE, CRF07_BC, and B subtypes are the three major subtypes in the MSM population [8]–[13]. Estimation of tMRCA for the three subtypes (CRF01_AE, CRF07_BC, B) in Chinese MSM clearly indicated different stages of HIV-1 transmission into MSM. The B subtype, which is the earliest circulating strain (around 1985) in the MSM population, is now declining. Not only that, US-derived B and Thai-B were first observed in Anhui and Henan MSM, which may be attributed to the local number of FBD infections with Thai-B. Notably, Thai-B and US-B subtype strains in MSM showed a closer relationship with MSM-derived B subtype strains from other epidemic regions rather than local circulating B strains in other high-risk populations (such as FBDs). These results clearly indicate a close genetic relationship among MSM strains.

CRF01_AE, entering into MSM in 2000, was the second earliest circulating strain and accounted for more than a half of the infections. CRF07_BC, entering into MSM in 2003, was the third entering strain, causing around 30% of the infection of MSM. As described in Figure 1D, the age of those infected with B subtypes was generally older than those infected with other subtypes, in accordance with the estimated introduction times of B subtypes into Anhui MSM. Combined with the timeline of different subtypes entering into MSM, the differing prevalence of the three subtypes in MSM further suggested some advantage in the newly entering strains that have been overtaking the B subtype in MSM. This was confirmed by observing dynamic changes in the prevalence rate of B, CRF01_AE, and CRF07_BC during 4 years of follow-up in Beijing MSM by Wang et al. [12], [13].

The MCMC trees of the three subtypes also showed significant transmission linkage among MSM strains from different epidemic regions: MSM strains from different epidemic regions always formed a close clade with high posterior probability. As mentioned above, the depicted epidemic information in Figure 1A and Table 2 also supported this close transmission linkage among MSM. Active transmission of HIV in Chinese MSM may facilitate the emergence of new recombinant strains. In this study, we identified several recombinant strains among subtype B, subtype C, CRF01_AE, and CRF07_BC in Anhui MSM. Particularly, 4 newly identified 01B recombinant stains, originating in Chinese MSM, showed distinct mosaic models with the isolated 01B strains from foreign countries, and are presumably well-adapted for transmission in the Chinese MSM population. Notably, the active transmission of HIV in MSM undoubtedly facilitates the transmission of emerging 01B recombinant strains in China, which may serve as a reminder of the importance of continuously monitoring the circulating strains in MSM.

As the main lab-force, the MSM are generally young and supposed to be competent in inducing immune response against invading pathogens. However, many patients with age less than 25 in Anhui MSM were diagnosed to be progressive into AIDS. Especially for the new recombinants infections, they all have low CD4 cell counts (<500 cells/µL) and more than 50% had obvious AIDS-like symptoms. This high AIDS progression rate calls for both further expanding investigation and early intervention therapy for the infected MSM.

Taken together, our studies not only characterized the virology and demographic characteristics of the Anhui MSM population, but also systematically revealed active transmission across the Chinese MSM population. The potential advantage and enhanced pathogenesis of emerging circulating recombinant strains, plus active transmission in MSM, make a new HIV pandemic possible in the Chinese MSM population.

## Supporting Information

Figure S1 **Bootscan analysis of 9** ***pol*** **partial gene sequences (HXB2, 2147–3462 nt).** 046, 059, 073, 115 had the similar breaking points of 01/B, and 64, 77 share another breakpoints of 01/B in *pol* gene region. 017, 069,104 displayed versatile breakpoints of CRF01_AE and CRF07_BC.
(TIF)

Figure S2 **The recombinant maps of two newly identified 01/B recombinant strains.** The recombinant maps were generated with Recombinant HIV-1 Drawing Tool (http://www.hiv.lanl.gov/content/sequence/DRAW_CRF/recommapper.html). Sequences 046, 059, 073 and 115 showed a similar recombinant structure while sequences 64 and 77 showed a different 01B recombinant pattern.
(TIF)
