# Supplementary figures and images for: New Emerging Recombinant HIV-1 Strains and Close Transmission Linkage of HIV-1 Strains in the Chinese MSM Population Indicate a New Epidemic Risk
Source: PLoS One. 2013 Jan 23;8(1):e54322. doi: 10.1371/journal.pone.0054322 (PMC3553145; doi:10.1371/journal.pone.0054322)

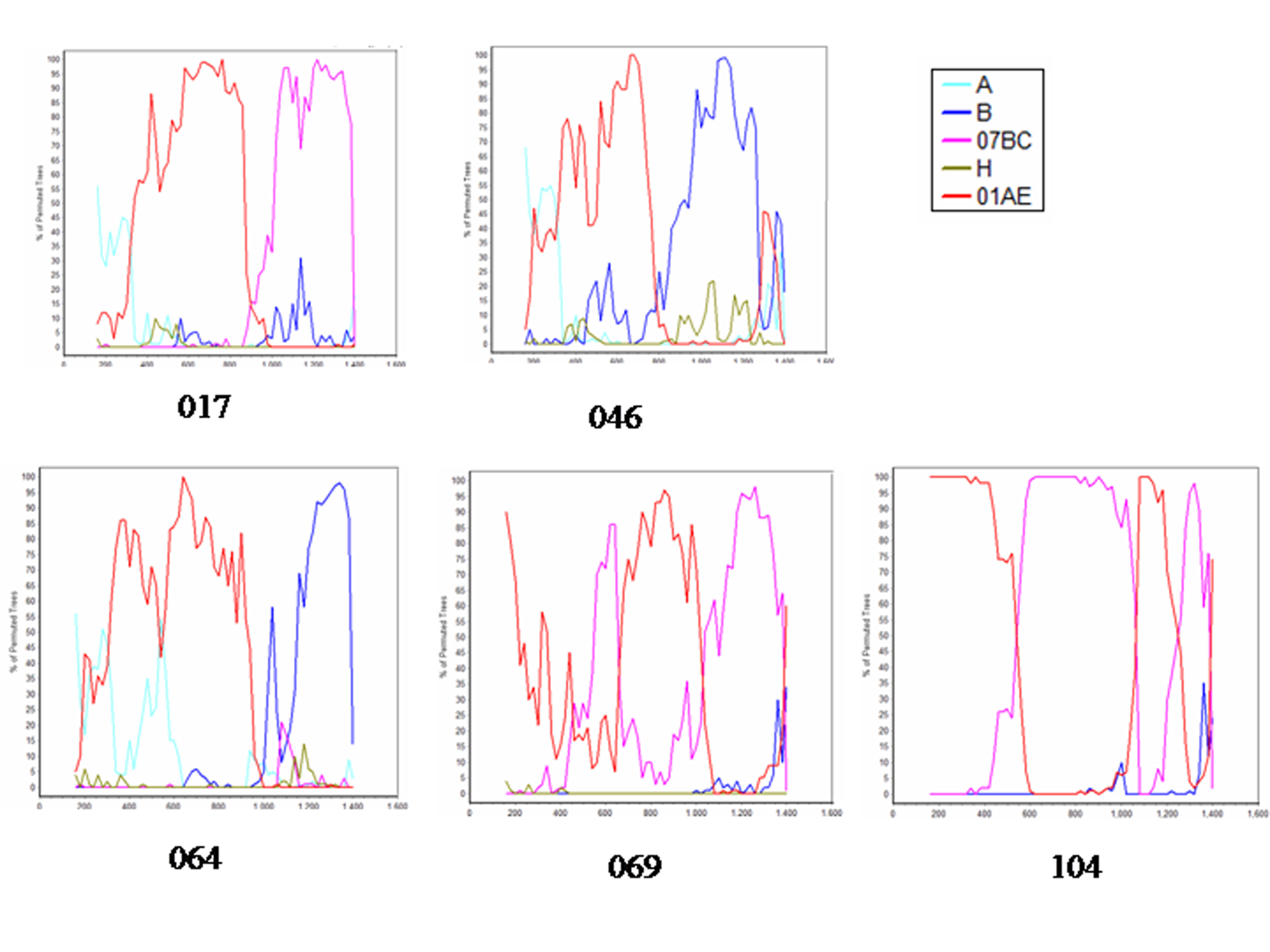

Supplement: Figure S1 — Bootscan analysis of 9 pol partial gene sequences (HXB2, 2147–3462 nt). 046, 059, 073, 115 had the similar breaking points of 01/B, and 64, 77 share another breakpoints of 01/B in pol gene region. 017, 069,104 displayed versatile breakpoints of CRF01_AE and CRF07_BC. (TIF) [file pone.0054322.s001.tif]

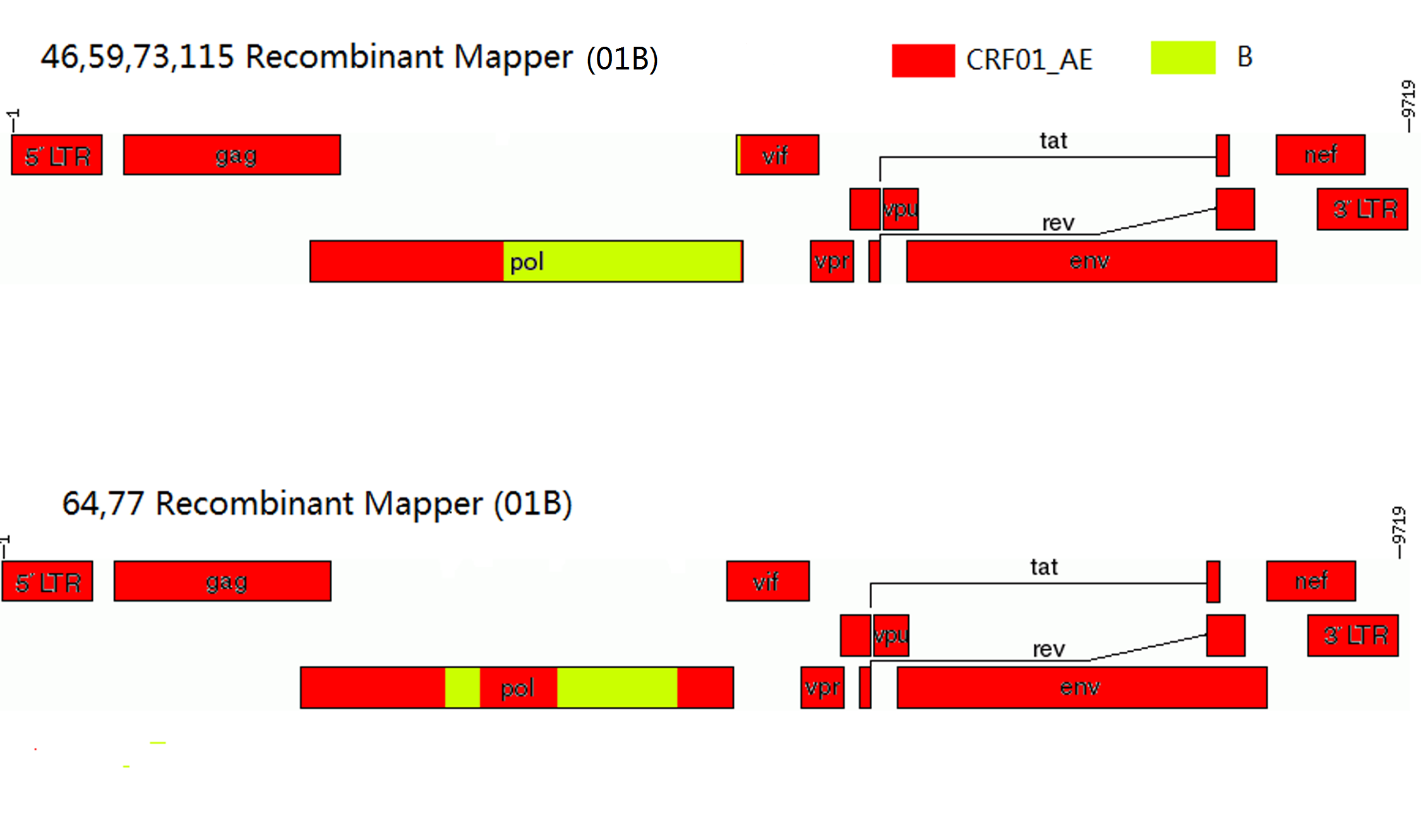

Supplement: Figure S2 — The recombinant maps of two newly identified 01/B recombinant strains. The recombinant maps were generated with Recombinant HIV-1 Drawing Tool (http://www.hiv.lanl.gov/content/sequence/DRAW_CRF/recommapper.html). Sequences 046, 059, 073 and 115 showed a similar recombinant structure while sequences 64 and 77 showed a different 01B recombinant pattern. (TIF) [file pone.0054322.s002.tif]
